# Apoptosis evasion via long non-coding RNAs in colorectal cancer

**DOI:** 10.1186/s12935-022-02695-8

**Published:** 2022-09-08

**Authors:** Muhammad Irfan, Zeeshan Javed, Khushbukhat Khan, Naila Khan, Anca Oana Docea, Daniela Calina, Javad Sharifi-Rad, William C. Cho

**Affiliations:** 1 Atta-Ur-Rahman School of Applied Biosciences, National University of Sciences and Technology, Islamabad, Pakistan; 2 Office for Research Innovation and Commercialization, Lahore Garrison University, Lahore, Pakistan; 3 Department of Toxicology, University of Medicine and Pharmacy of Craiova, 200349 Craiova, Romania; 4 Department of Clinical Pharmacy, University of Medicine and Pharmacy of Craiova, 200349 Craiova, Romania; 5 Facultad de Medicina, Universidad del Azuay, Cuenca, Ecuador; 6 Department of Clinical Oncology, Queen Elizabeth Hospital, Kowloon, Hong Kong

**Keywords:** Colorectal cancer, Long Non-coding RNA, Apoptosis, Epigenetic, miRNA

## Abstract

Long non-coding RNA (LncRNA) is a novel and diverse class of regulatory transcripts that are frequently dysregulated in numerous tumor types. LncRNAs are involved in a complicated molecular network, regulating gene expression, and modulating diverse cellular activities in different cancers including colorectal cancer (CRC). Evidence indicates that lncRNAs can be used as a potential biomarker for the prognosis and diagnosis of CRC as they are aberrantly expressed in CRC cells. The high expression or silencing of lncRNAs is associated with cell proliferation, invasion, metastasis, chemoresistance and apoptosis in CRC. LncRNAs exert both pro-apoptotic and anti-apoptotic functions in CRC. The expression of some oncogene lncRNAs is upregulated which leads to the inhibition of apoptotic pathways, similarly, the tumor suppressor lncRNAs are downregulated in CRC. In this review, we describe the function and mechanisms of lncRNAs to regulate the expression of genes that are involved directly or indirectly in controlling cellular apoptosis in CRC. Furthermore, we also discussed the different apoptotic pathways in normal cells and the mechanisms by which CRC evade apoptosis.

## Introduction

Colorectal cancer (CRC) is the second most common cancer after lung cancer in adults. The varied incidence between and within countries suggests that numerous etiological factors like genetic, epigenetic, and environmental factors may be involved in the development and progression of CRC [1, 2]. It starts in the form of benign adenomatous polyp which grows into a complex adenoma with high-grade dysplasia and then eventually into invasive cancer. The ability of CRC to spread to other organs of the body leads to a higher mortality risk. It is therefore the need today to investigate the molecular mechanism in the development and progression of CRC so that new therapeutic strategies can be developed [3, 4]. In the past, a significant amount of research has been done on the protein-coding genes and epigenetic changes contributing to colorectal tumor initiation and progression, but the discovery and documentation of long non-coding RNAs (lncRNAs), has added a new level of complexity to the molecular landscape of CRC [5, 6].

LncRNAs occupying about 70% of the human genome are polyadenylated transcripts with length > 200 nt, that are not translated into proteins [7, 8]. Research shows that dysregulation of certain lncRNAs is involved in diverse cellular processes including proliferation, differentiation and death of cancer cells [9]. The lncRNAs control gene expression at both transcriptional and post-transcriptional levels. The mechanism of regulating gene expression depends upon the subcellular localization of lncRNAs [10]. The nuclear lncRNA interacts with DNA at specific loci recruiting epigenetic modifying complexes and affecting chromatin structure [11]. On the other hand, cytoplasmic lncRNAs gene regulation occurs at the post-transcriptional level mostly by serving lncRNA as competing for endogenous RNA (ceRNA) binding with specific miRNA molecules and preventing them to bind with target mRNA [12].

A significant amount of research demonstrated the dysregulated expression of lncRNAs in CRC, with one study identifying about 200 differentially expressed lncRNAs [13]. Emerging evidence suggests that the aberrant expression of lncRNAs plays a vital role in colorectal cancer by modulating important processes including metastasis, chemoresistance and cellular apoptosis LncRNAs target both intrinsic and extrinsic pathways of apoptosis in CRC. The abnormalities in apoptotic pathways that are driven by lncRNAs contribute to the pathogenesis of CRC and its resistance to therapeutic drugs [14].

In this review, we aimed to summarize the current understanding of the functions and mechanisms of lncRNAs involved in modulating apoptotic pathways in colorectal cancer. The apoptotic pathway is deregulated in many cancers including CRC and thus effective therapies designed to stimulate this process in target cells would play a significant role in controlling the development and progression of CRC.

## Methodology

For this comprehensive review, studies were conducted that highlighted the role of lncRNAs involved in modulating apoptotic pathways in colorectal cancer. Databases such as PubMed/Medline, Google Scholar, Scopus and UpToDate were searched using the following MeSH terms: MeSH terms: “Apoptosis”, “Cell Movement”, “Cell Proliferation”, “Cell Transformation, Neoplastic”, “Colorectal Neoplasms/genetics”, “Colorectal Neoplasms/ pathology”, “Gene Expression Regulation”, “Neoplastic”, “Humans”, “RNA, Long Noncoding/genetics”. The selected studies were evaluated to find the most important data on colorectal cancer apoptosis, related signaling pathways, molecule targets, functions and roles of lncRNAs as well as cell line characteristics included in the analyzed studies. The most significant data were summarized in tables and the molecular mechanisms were schematically represented in figures.

## Apoptosis and evading apoptosis in CRC

**Fig. 1:**
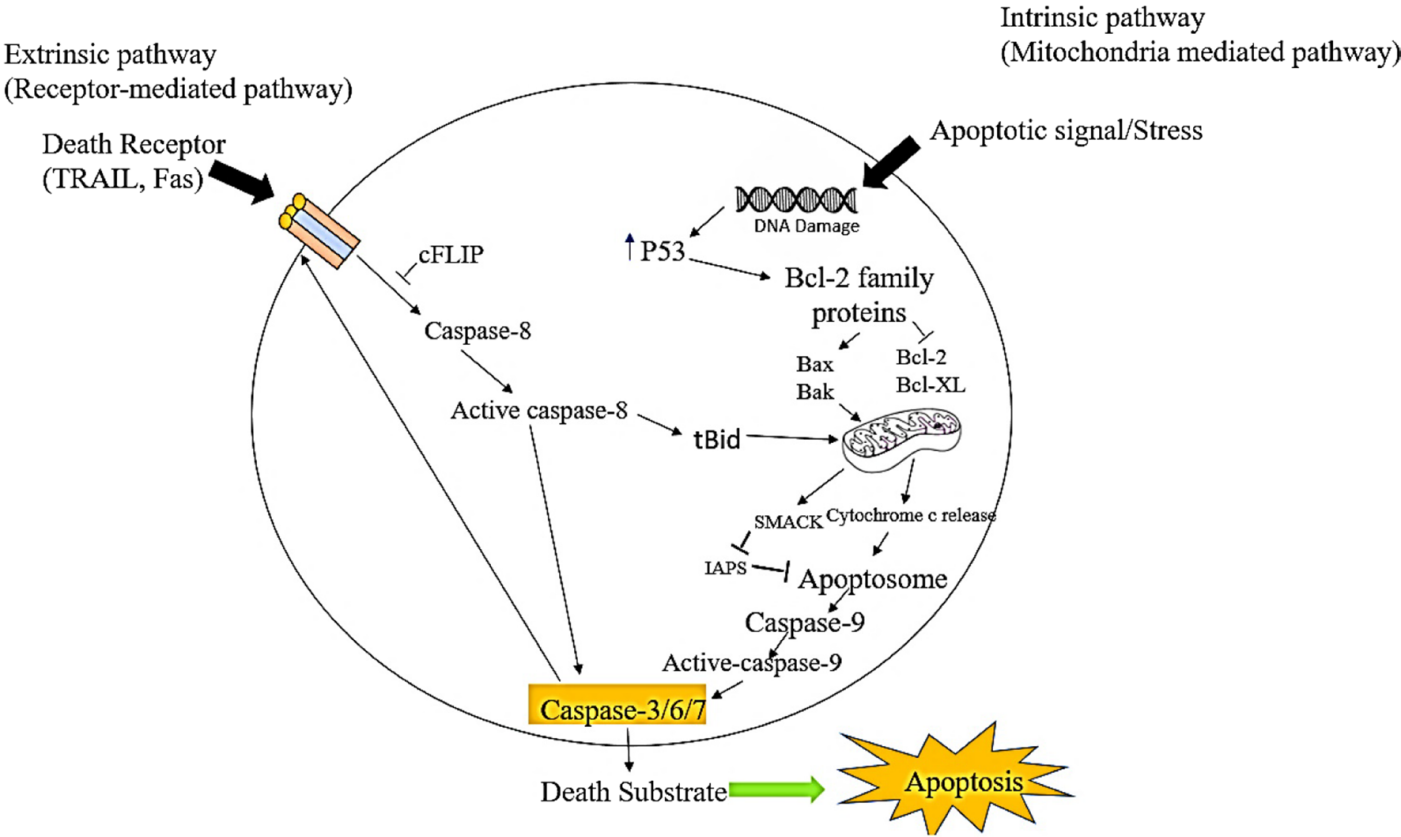
Apoptotic pathways in a normal cell. The extrinsic pathway is activated by the attachment of the extracellular ligand to death receptors (DRs). The intrinsic pathway is initiated by Bax/Bak incorporation into mitochondrial membrane followed by cytochrome c release which then along with Apaf–1 and procaspase-9 make apoptosome. Activation of the caspase 3 cascade of apoptosis takes place after apoptosome formation. (Key: TRAIL (TNF-related apoptosis-inducing ligand), cFLIP (cellular FLICE inhibitory proteins), tBid (Truncated bid), Bcl-2 (B-cell lymphoma protein 2), Bcl-xL (Bcl-2 homologue splice variants), Cyt C (Cytochrome), SMAC (Second mitochondrial activator of caspases), IAPs (Inhibitor of apoptosis proteins)

A programmed cell death pathway that facilitates eliminating cells that are no longer needed or have severe damage to their DNA and cytoskeleton is known as apoptosis [15–17]. The mechanism of apoptosis is predominantly induced by two core pathways: the extrinsic (Death receptor-mediated) pathway and the intrinsic (mitochondrial-mediated) pathway (Fig. 1). Both these pathways in the end lead to cell death [18].

### Intrinsic pathway

The activation of the intrinsic pathway is initiated by both internal and external stimuli. These stimuli can be generated as a result of any injury, DNA damage, oncogenes, cytotoxic drug treatment, insufficient survival factors and hypoxia etc. [19, 20]. The Bax/Bak are the major proteins that mediate the intrinsic pathway. Their entry into the mitochondrial membrane is followed by the release of cytochrome c into the cytosol. On the other hand, Bcl-2 and Bcl-xl prevent the intrinsic pathway via inhibiting cytochrome c to release as they are anti-apoptotic proteins [21]. The binding of cytochrome c with Apaf-1 and procaspase-9 results in a multi-complex protein called the apoptosome, which stimulates caspase-9 and the subsequent activation of the caspase-3 signalling cascade [22]. This signalling results in cell annihilation and leads to apoptosis. SMAC/DIABLO (Second mitochondrial activator of caspases/direct IAP binding protein with low PI), Aven (Cell death regulator Aven), Bcl-w (Bcl-2 like protein), Caspase-9 (Cysteinyl aspartic acid-protease-9), Bcl-2 (B-cell lymphoma protein 2), Nox (Phorbol-12-myristate-13-acetate-induced protein 1) and Myc (Oncogene Myc) are some of the proteins that play important role in the intrinsic pathway [23]

### Extrinsic pathway

The extrinsic pathway is initiated by the attachment of extracellular growth factors i.e., TNF (tumor necrosis factor), Fas-L (Fas ligand), and TRAIL (TNF-related apoptosis-inducing ligand) with the DR (transmembrane receptors) i.e. type 1 TNF receptor (TNFR1), Fas (also called CD95/Apo-1) and TRAIL’s extracellular domain [24, 25]. The binding of DLs (Death ligands) with DRs leads to death-inducing signalling complex (DISC)[26] formation and activation. DISC comprised of procaspase-8, procaspase-10 and Fas-associated death domain act as an adopter molecule and cellular FLICE inhibitory proteins (c-FLIPs). The detachment of active caspase-8 from its pro-domain which remain on the DISC results in the activation of executioner caspases and leads to the execution pathway of apoptosis.

### Execution pathway

Both intrinsic and extrinsic pathways share the final phase or the execution phase of apoptosis [27, 28]. The caspases involved in apoptosis are distinguished into initiator caspases (Caspase-8 and 9) and executioner caspases i.e., caspase-3, caspase-6 and caspase-7, Caspase-10, CAD (Caspase-activated DNAse) and PARP (Poly (ADP-ribose) polymerase) [29, 30]. Initiator caspases are activated in an autocleavage manner that subsequently activates the executioner caspases. These caspases are involved in the proteolysis of substrates resulting in cell death. The difference between initiator caspases and executioner caspases lies in their pro-domain as the initiator has a long pro-domain having an adopter molecule involved in multimerization in contrast to the executioner with a short pro-domain. These effector/executioner caspases can lead to cytoplasmic blebs, apoptotic bodies formation and condensation of chromatin [31]. The activation of effector caspases is the onset of this pathway, followed by the activation of cytoplasmic endonuclease, which lead to nuclear material and protease degradation and proteolysis of nuclear and cytoskeletal proteins. Caspase-3 is activated by any of the initiator caspases which makes it the most potent and important executioner caspase. CAD endonuclease, involved in chromosomal DNA degradation and chromatin condensation, is readily activated by caspase 3 [19, 32].

### Apoptosis evasion in CRC

The homeostasis in the intestine is regulated by the tight balance of cell division and programmed cell death (apoptosis) [33]. The disturbance in this balance is one of the major steps involved in the initiation and progression of colorectal cancer. The apoptosis cascades act as an anti-cancer defence mechanism and the evasion of these mechanisms to bypass cell death allows uncontrolled proliferation in many cancers including CRC [34–37] (Table 1). One of the mechanisms by which cancer evades apoptosis is to increase the apoptotic threshold through modulating either the expression or the activity of Bcl-2 family proteins. This protein family include both pro-and anti-apoptotic proteins, and the proper interaction of these proteins determines if the cell will undergo proliferation or apoptosis [38]. The deregulation of Bcl-2 family proteins which leads to cell survival and resistance is frequently observed in tumor cells [39, 40].

**Table 1:** Up/down regulation of apoptotic genes in different cancer types

Pro/antiapoptotic gene	Outcome	Cancer type	Ref.
FAS	Downregulation	T-cell lymphoma, colon carcinoma, neuroblastoma, melanoma, ovarian cancer	[62–66]
DR4/DR5	Downregulation	Medulloblastoma	[67]
CASPASE-8/10	Downregulation	Hepatocellular carcinoma, bladder, small-cell lung carcinoma, GBM, retinoblastoma, and neuroblastoma	[68–72]
BIM	Downregulation	Renal cell carcinoma and chronic myeloid leukemia Burkitt’s lymphoma	[73, 74]
APAF-1	Downregulation	Leukemia, melanoma, and gastric, bladder, and kidney cancer	[75–78]
XAF1	Downregulation	Gastric and bladder cancer, Prostate cancer, Pituitary adenoma, B-cell chronic lymphocyte leukemia	[79–84]
BCL-2	Downregulation	Gastric cancer, chronic lymphocytic leukemia, pancreatic, breast, colon, and kidney cancer, and Burkitt’s lymphoma	[85, 86]
BAX	Downregulation	Multiple myeloma cells, Burkitt’s lymphoma, colon cancer	[87–89]
BAK	Downregulation	Multiple myeloma cells, Burkitt’s lymphoma	[87, 88]
PUMA	Upregulation	Multiple myeloma cells, Burkitt’s lymphoma	[87, 88]
BAD	Upregulation	Multiple myeloma cells	[87]
Bcl-2L10	Downregulation	Gastric cancer and leukemia	[90, 91]
BIK	Downregulation	Glioma, RCC, prostate cancer, and myeloma	[92–95]
BNIP3	Downregulation	Gastric cancer, colorectal cancer, leukemia, and HCC	[96–99]
HRK	Downregulation	Colorectal, gastric, GBM, PCNSL, and prostate cancer	[100–103]
HRK	Upregulation	Lung, prostate, bladder, and ovarian cancers and GBM	[104–108]
MLC1	Upregulation	AML, gastric tumors, GBM, and lung cancer	[109–112]
BCL-6	Upregulation	Bladder, prostate, breast, and lung cancer and lymphoma	[113]
PUMA CASPASE-3	Downregulation	Bladder and glioma	[114, 115]
Bcl-XL	Upregulation	Hodgkin lymphoma, AML, and ovarian cancer	[116, 117]
Bcl-2	Upregulation	Gastric cancer	[118]

**Fig. 2:**
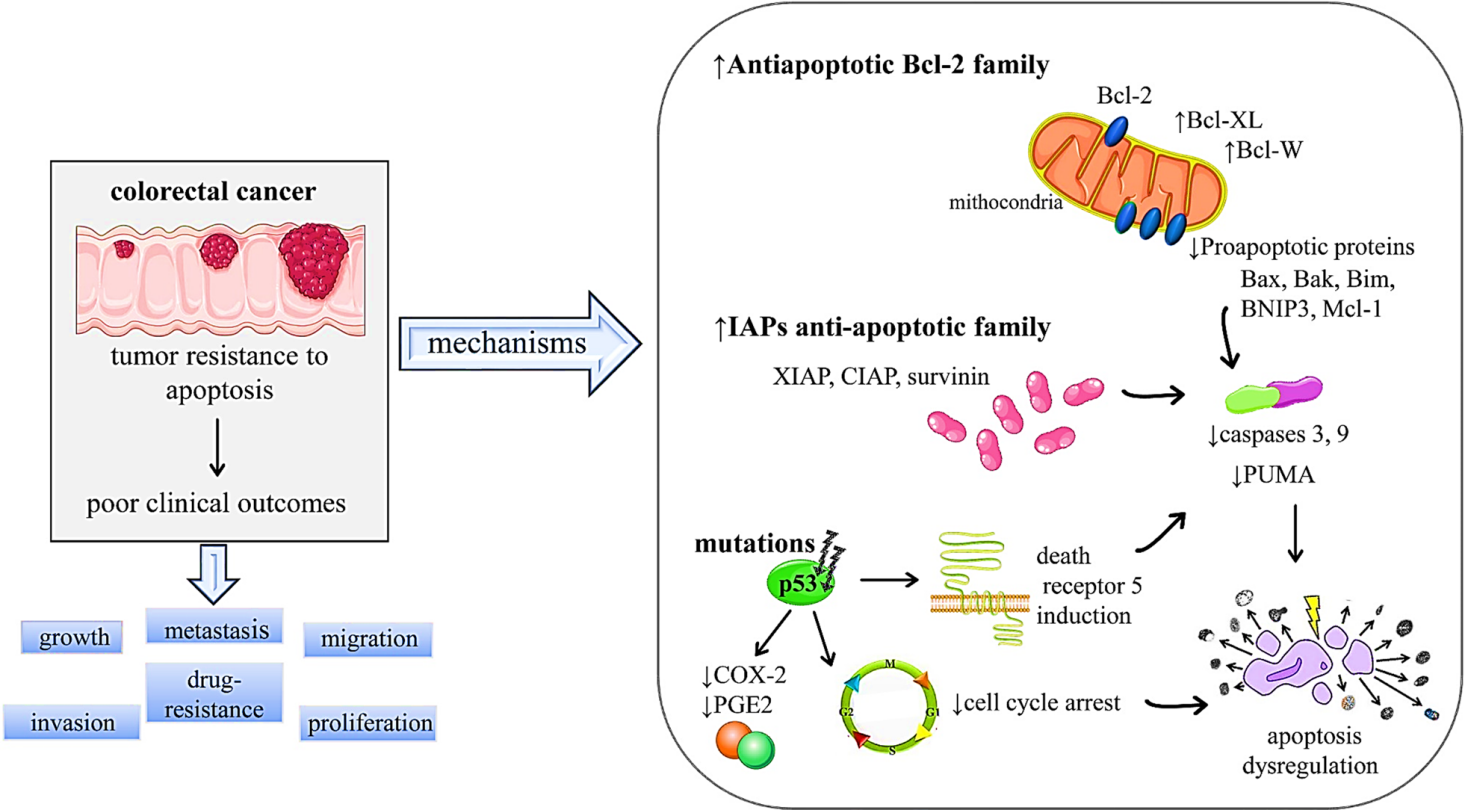
Summarized scheme with mechanisms of apoptosis evasion in colorectal cancer. Symbols: ↓down-regulation, ↑ up-regulation. Abbreviations and symbols: ↑ (increase), ↓ (decrease), Bcl-2 (B-cell lymphoma 2), Bcl-XL (B-cell lymphoma-extra large), Bax (BCL2 Associated X, Apoptosis Regulator), Bim (B cell lymphoma-2-like 11), MCL-1 (Myeloid cell leukemia-1), \XIAP **(**X-linked inhibitor of apoptosis protein), CIAP (cellular inhibitor of apoptosis), PUMA (p53 upregulated modulator of apoptosis), COX-2 (cyclo-oxygenase-2), PGE2 ( Prostaglandin E2)

A significant number of studies have reported the upregulation of Bcl-XL a member of the Bcl-2 family in CRC tumor cells as compared to normal cells, where it is important for proliferation and resistance [40–42]. Bcl-W another anti-apoptotic Bcl-2 family member is also associated with progression from adenoma to adenocarcinoma. The overexpression of both Bcl-XL and Bcl-W is found to be associated with the downregulation of Bax a pro-apoptotic gene in primary colorectal adenocarcinomas [42, 43]. Similarly, the overexpression of Mcl-1 is also an indicator of poor outcomes in colorectal cancer [44], as loss of Mcl-1 was found to be associated with increased expression of caspase- 3, 9 and also PUMA which is a proapoptotic protein [45, 46]. Mutations and downregulation of proapoptotic genes such as Bax, Bak, Bim and BNIP3 also render the cells resistant to apoptosis and are associated with poor prognosis in colorectal cancer [47–51] (Fig. 2).

The p53 the best-known tumor suppressor is involved in the mitochondrial apoptotic pathway. It is an important protein that regulates the cell cycle and controls programmed cell death [52, 53]. The mechanisms by which p53 causes apoptosis include the regulation of PUMA expression, free radicals generation within mitochondrial components, reduction of COX-2, PGE2 synthesis and the induction of death receptor 5 [54] The mutation in p53 leads to oncogenesis, and about half of all colorectal cancers are associated with Tp53 mutations [55] (Fig. 2). Another family of proteins called inhibitors of apoptosis (IAPs) also causes resistance to apoptosis mostly by inhibiting effector caspases [16, 56, 57]. In colorectal cancer, the expression of IAPs is disturbed which leads to apoptosis suppression. The anti-apoptotic members of this family including XIAP, cIAP and survivin bind to caspase 3 and 9 inhibiting their activity. The overexpression of these proteins is upregulated in colorectal cancer and is associated with the progression of the disease and poor survival [58, 59]. Furthermore, the downregulation of caspases such as caspase -3 and 9 is also responsible for apoptosis suppression and poor clinical outcomes in colorectal cancers [60, 61] (Fig. 2).

## The crosstalk between LcRNAs and apoptosis in CRC

**Fig. 3:**
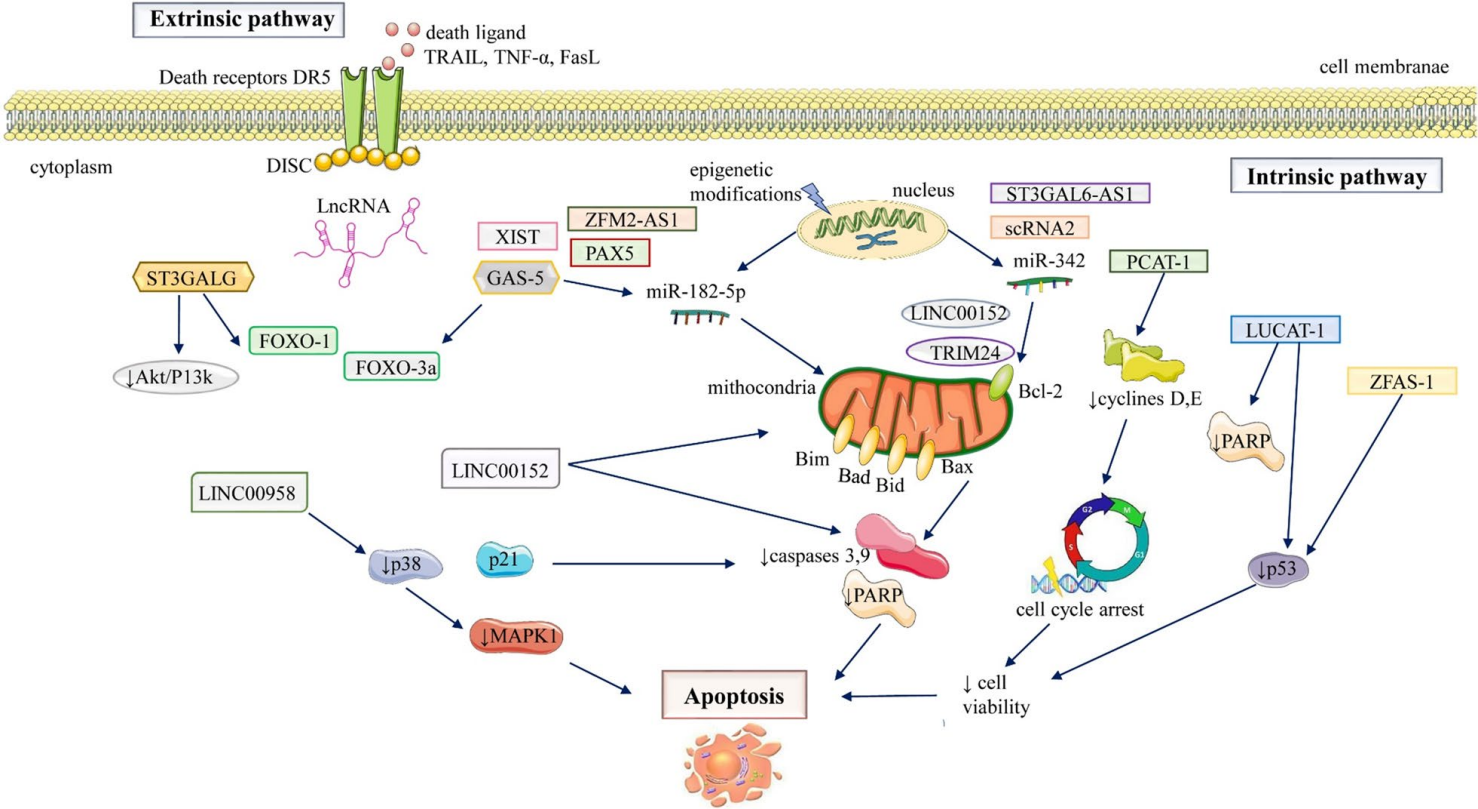
Diagram with apoptosis mechanisms in colorectal cancer. This is mediated via intrinsic (mitochondrial) or extrinsic pathways including TRAIL, FasL and TNF signaling cascades that by interacting with their respective receptors, result in the activation of caspases

Evidence demonstrating the pro-apoptotic contribution of lncRNA in CRC is reported. LncRNAs interact with several upstream and downstream effecter molecules of apoptotic pathways [119–121]. The expression of oncogene lncRNAs is up-regulated which also boosts pathways that facilitate inhibition of apoptotic pathways [122, 123]. Similarly, tumour-suppressive lncRNAs expression is down-regulated that promoting the expression of anti-apoptotic components. A list of lncRNAs that directly or indirectly control the expression of apoptotic pathway components in CRC along with their target and regulatory mechanism is given in Table 2 and Fig. 3.

**Table 2:** List of TRAIL pathway-specific lncRNAs along with their adopted mechanism

LncRNA	Expression	Comment	Ref
ST3GAL6-AS1	Down-regulated	Indirect regulation of FasL, TRAIL ligand, Bad, Bim	[141, 142]
LINC00460	Up-regulated	↓TRAIL, ↓caspase-9, ↓Bax, ↓Bcl-2	[149]
GAS5	Down-regulated	↑TRAIL l, ↑FasL, ↓miR‑182‑5p	[143, 144]
LINC00152	Down-regulated	↓Bcl-2, ↑ FasL	[146]
PCAT-1	Up-regulated	↓PARP, ↓caspase-3 cleavage, ↓Bax, ↑Bcl-2	[128]
SCARNA2	Up-regulated	↑Bcl-2, ↑EGFR expression via miR-342-p sequestering	[124]
HAGLROS	Up-regulated	↑autophagy, inhibition of extrinsic apoptotic pathway ↑ATG5	[160]
LUCAT1	Up-regulated	dgradation of p53 at protein level	[129]
ZFAS1	Up-regulated	↑p54 destabilization, ↓PARP cleavage	[131]
MAPKAPK5‐AS1	Up-regulated	Inhibition of extrinsic apoptosis via epigenetic silencing of p21	[163]
HOXA-AS2	Up-regulated	↓p21 transcription	[164]
BANCR	Down-regulated	↑ p21 translation	[165]
ATB	Up-regulated	↓p2, ↓p53 expression via regulation of miR-200c	[168]
FOXP 4‐AS1	Up-regulated	↓ p21	[166]
SNHG6	Up-regulated	epigenetic silencing of p21	[167]

### Intrinsic apoptosis (mitochondrial pathway)

Most identified lncRNA modulates TRAIL-mediated apoptosis by either promoting or suppressing the expression of Bcl-2 which then brings about intense tumor growth, enhanced metastatic ability and chemoresistance. LncRNA Cajal body-associated RNA2 (scaRNA2) expression is significantly up-regulated in CRC and is reported to be associated with larger tumor size, chemoresistance and metastasis. Evidence demonstrated that sca RNA2 induces tumor proliferation by promoting the expression of Bcl-2 and EGFR [124] (Fig. 3).

Grady et al. reported tumor-suppressive properties of miR-342, the expression of which is suppressed by epigenetic modification [125]. The expression of scaRNA2 is inversely associated with the expression of miR-342. According to Lai et al., miR-342 promotes apoptosis by inhibiting the expression of Bcl-2 and inducing the expression of Bax while the reduced expression of miR-342 causes CRC cells’ resistance to chemotherapy by boosting the anti-apoptotic pathway [126]. So, scaRNA2 induces chemo-resistance and exerts anti-apoptotic influence by suppressing miR-342 and consequently enabling the expression of Bcl-2 [124]. These findings indicate that scaRNA2 is an excellent therapeutic target for curbing tumor growth in advanced stages of CRC. Moreover, targeting scaRNA2 can also enhance the anti-cancer effects of practised drugs whose influence has been lost due to developed chemoresistance.

Similarly, lncRNA prostate cancer-associated transcript 1 (PCAT-1) is also highly expressed in CRC tissues and its cancer cell lines. Its elevated expression has a positive correlation with CRC tumor progression, metastasis and poor survival [127]. Qiao et al. inhibited its expression in Caco-2 and HT-29 cells by treating these cell lines with PCAT-1 sequence complementary shRNA2-PCAT-1. They found that reduction in PCAT-1 expression caused a decrease in cyclin D1 and cyclin E levels which led to cell cycle arrest and growth inhibition. Further by performing Hoechst staining and flow cytometry, they determined that the rate of apoptosis was significantly raised after treatment with shRNA2-PCAT-1. Its down-regulation induced apoptosis by promoting the expression of Bax and cleavage of PARP and caspase-3 along with suppressing the expression of Bcl-2 [128].

LncRNA lung cancer‐associated transcript 1 (LUCAT1) exerts its modulatory influence on the apoptotic pathway by inhibiting the expression of PARP-1. Its expression is up-regulated in CRC and has a direct correlation with poor prognosis and advanced tumor stages. LUCAT1 in vitro over-expression brings about rapid tumor proliferation and invasiveness. Knocking down of LUCAT1 caused a reduction in cell viability and induced cell growth arrest. Moreover, LUCAT1 silencing also facilitated apoptosis by promoting the expression of PARP-1. Zhou and the group demonstrated the association of LUCAT1 with inhibition of p53 signaling. They reported that LUCAT1 knock-down enhanced the stability of p53 protein which consequently brought about tumor cell apoptosis. LUCAT1 interacts with UBA52 and together induces degradation of p53 and hence halts tumor cell apoptosis [129] (Fig. 3).

The findings of Thorenoor et al. demonstrated the oncogenic properties of lncRNA zinc finger antisense 1 (ZFAS1) which promotes cell progression by destabilizing p53. It is highly expressed in CRC tissues and HCT-116, HT-29 and SW-620 CRC cell lines and is responsible for tumor metastasis and poor prognosis [130]. They blocked ZFAS1 transcription via siRNA treatment and found that down-regulation of ZFAS1 attenuated colony-forming ability, induced cell cycle arrest, halted cell progression and promoted apoptosis by enhancing PARP cleavage, increasing p53 expression levels and reducing cyclin B1 levels [131] (Fig. 3). The high expression of LINC00908 an oncogenic lncRNA was reported in CRC tissues and cells. It was found that the knockdown of LINC00908 in CRC cells decreased the expression of proteins like cyclin D1, CDK4, Rb, and p-Rb and thus inhibited the proliferation of CRC cells. Furthermore, the flow cytometry analysis revealed that the inhibition of cell growth was partly due to the induction of apoptosis that occurred due to LINC00908 silencing. The mechanism of LINC00908-induced apoptosis was investigated, and it was observed that the downregulation of LINC00908 initiated the intrinsic apoptotic pathway by enhancing the cleavage and thus increasing the levels of caspase-9 and caspase-3 [132].

Zhang et al. investigated the correlation between the expression of X-inactive specific transcript (XIST) an important lncRNA and the clinicopathological features of CRC. The study revealed that the expression level of XIST was highly upregulated in CRC and was found to be correlated with enhanced proliferation and poor survival [133]. lncRNA X-inactive specific transcript(XIST) modulates multiple processes including cell proliferation, metastasis and apoptosis in colorectal cancer by acting as a miRNA sponge. The silencing of XIST leads to decreased cell proliferation, and migration and induces apoptosis. The increased expression of XIST downregulates the miR-338-3p by directly binding with it and in turn increases the expression of the pair-box gene (PAX5) a downstream target of miR-338-3p. These findings demonstrate that XIST acts as an oncogene and can be used as a potential target for the diagnosis and treatment of CRC [134]. In addition, the elevated levels of another lncRNA FOG family member 2 antisense RNA 1 (ZFPM2-AS1) were demonstrated to be correlated with cell proliferation, migration, and invasion in CRC. The ZFPM2-AS1 acts as competing endogenous RNA (ceRNA) and competitively binds with miR-137 and in turn, upregulates the expression of tripartite motif-containing 24 (TRIM24) which is a downstream target of miR-137 [135]. Previous studies showed that the upregulation of TRIM24 is associated with increased proliferation and inhibition of apoptosis in CRC cells, Moreover, the knockdown of TRIM24 leads to a decrease in the expression of Bcl-2 and a significant increase in the expression of caspase 3 and PARP [136, 137].

Recently the expression profiles of a novel lncRNA LINC02474 were studied to investigate its role in modulating metastasis and apoptosis in colorectal cancer cells. The study showed that LINC02474 acts as an oncogene and its elevated levels are associated with intensified invasion and reduced apoptosis. The LINC02474 limits apoptosis in CRC by reducing the expression of Granzyme B (GZMB), which is an enzyme with cell-killing abilities secreted by cytotoxic T lymphocytes and natural killer cells [138]. The GZMB contribute to apoptosis by acting as a serine protease and hydrolytically cleaving caspase-3 and Bid to activate them [139].

### Extrinsic apoptosis

Extrinsic apoptosis includes TRAIL, FasL and TNF signaling cascades that by interacting with their respective receptors, resulting in the activation of caspases. LncRNAs interact with extrinsic apoptotic pathways at different stages that eventually cause inhibition of signal transduction through these pathways.

#### Ligand/Receptor modulation

Initiation of extrinsic apoptotic pathway takes place with ligands such as TRAIL, FasL or TNF binding with their death receptors. LncRNA ST3GAL6 Antisense RNA 1 (ST3GAL6-AS1) gene lower expression was reported in highly metastatic cell line SW620 of CRC. It is mapped on chromosome 3q11.2 and is transcribed from the promoter region of sialyltransferase ST3Gal6 [140]. It indirectly modulates TRAIL signaling by inhibiting Akt/PI3K signaling and inducing nuclear translocation of FOXO-1 [141]. It is then FOXO-1 that facilitates apoptosis in CRC cells by bringing about transcription of FasL, Bim, BAD and death receptor ligand, TRAIL [142]. The over-expression of 769 nucleotide long lncRNA ST3GAL6-AS1 is negatively associated with tumor stage, size and metastasis in CRC patients which makes it a potential diagnostic marker for this carcinoma [141].

LncRNA growth arrest-specific 5 (GAS5) modulates apoptosis in CRC by regulating FOXO3a expression. Similar to FOXO-1, FOXO3a also promotes apoptosis by induction of FasL and TRAIL ligand expression [143]. The expression of GAS5 is down-regulated in CRC tumor tissues and HCT-116, SW480, HT-29 and LoVo cell lines which are associated with lymph node metastasis and advanced clinical stages of cancer. Cheng and colleagues induced the expression of GAS5 by transfection of pcDNA3.1-GAS5 plasmids in CRC cell lines and reported attenuated cell proliferation and increased apoptosis rate in CRC cells [144]. The expression of FOXO3a is regulated by miR‑182‑5p in different cancers including CRC. So mechanistically, GAS5 promotes apoptosis by inhibiting the expression of miR‑182‑5p and up-regulating FOXO3a expression [144, 145].

The aberrant reduction in expression of lncRNA LINC00152 in colon cancer cell lines was observed by Zhang et al. They reported that the lentivirus-based transient expression of LINC00152 in SW480 and HT-29 CRC cell lines reduced the viability of cancerous cells and simultaneously promoted apoptosis by up-regulating the expression of FasL and down-regulating expression of Bcl-2 [146]. Contrarily, Bian et al. found high expression of LINC00152 in CRC tumor tissues and reported its growth-stimulating functions along with its association with poor prognosis [147]. Their findings were in accord with Chen and groups who also reported elevated LINC00152 expression in HT29 and HCT116 cell lines. They also reported the association of LINC00152 with cancer cell migration and invasiveness [148]. The link between LINC00152 and chemo-resistance is also demonstrated by Bian et al*.* and Chen et al. According to them, knocking down LINC00152 not only reduced cell invasiveness but also sensitized cells to chemotherapy [147, 148]. These findings high lights LINC00152 as a potential biomarker for CRC but the contradictory outcomes of Zhang et al*.* [146] stress the need for further research to explore LINC00152 carcinogenic potential.

Lian and group identified lncRNA LINC00460 through CRC microarray profile analysis which is 935 nucleotides long and is mapped on chromosome 13q33.2. They reported that LINC00460 high expression has a direct correlation with tumor proliferation in CRC cell lines: lovo, SW480, HCT116 and HT-29. While it’s in vivo over-expression is associated with advanced tumor stages, metastasis in lymph nodes and shorter survival. Their experimentation further revealed that knocking down of LINC00460 via complementary siRNA treatment induces activation of caspase 3. Moreover, its up-regulated expression in CRC tissues down-regulates Trail, caspase-9, Bax and Bcl-2 expression [149]. This finding suggests that LINC00460 halts TRAIL-mediated apoptosis by suppressing the expression of TRAIL ligand and downstream factors of this pathway. But whether it directly suppresses TRAIL expression or vice versa is still needs to be determined.

Several evidences have established that the p38 pathway plays a significant role in TRAIL-induced apoptosis. P38 brings about transcription of TRAIL and DR5 and eventually, participates in cell apoptosis [150, 151]. But in some cancer cell lines including HT-29, HCT-116 and DLD-1 colon cancer cell lines, the role of p38 is associated with cell growth and proliferation [152, 153]. Li et al. detected lower expression of lncRNA SLC25A25-AS1 in tumor tissues of CRC patients and several CRC cell lines. They reported that SLC25A25-AS1 regulates phosphorylation activation of p38 and its under-expression causes chemo-resistance. They further demonstrated in vitro that induced expression of SLC25A25-AS1 inhibited p38 activation and had a direct correlation with suppressed cell proliferation and attenuated colony-forming ability [153].

The expression of LINC00958 an oncogene lncRNA was found to be upregulated in colorectal cancer as compared to the normal tissues. The overexpression of this lncRNA significantly decreased apoptosis, promoted proliferation and suppressed radiosensitivity in colorectal cancer. The possible mechanism by which LINC00958 suppress apoptosis in colorectal cancer is that it acts as a miR-422a sponge and stops its inhibition of MAPK1 expression, which is involved in several biological processes including apoptosis [154, 155].

#### Adaptor proteins and DISC regulation

Autophagy is a cell’s catabolic process to degrade its cytoplasmic components in lysosomes and is necessary for the maintenance of cell homeostasis and cell survival [156, 157]. Several studies have shown the participation of TRAIL in inducing autophagy. In TRAIL-induced autophagy, ATG5 plays a key role. Its interaction with FADD interrupts the formation of the FADD/DISC complex and hence, inhibits extrinsic apoptosis [158, 159]. A study has demonstrated the role of lncRNA HAGLROS in inducing autophagy and inhibiting apoptosis in the HCT-116 CRC cell line. Its elevated expression is reported in CRC which has a positive correlation with shorter survival. HAGLROS facilitates autophagy by promoting the expression of ATG5 [160]. ATG5 is a direct target for miR-100, so HAGLROS suppresses miR-100 which allows translation of ATG5 mRNA [160, 161]. Zheng and colleagues demonstrated by knocking down HAGLROS in HCT-116 that inhibition of HAGLROS inhibited autophagy and activated TRAIL-induced apoptosis. HAGLROS inhibition brought about miR-100-induced suppression of ATG which furthered the activation of caspase-3 and -9 along with elevating the Bax expression [160].

#### Caspases modulation

TRAIL-mediated extrinsic apoptosis is regulated by p21 which is a cell-cycle regulatory molecule. Although it inhibits apoptosis by inhibiting caspase 3 activation some studies do indicate a pro-apoptotic role of p21. In which case, its cleavage via caspase 3 leads to apoptosis [162]. In CRC cell lines SW480 and DLD-1, lncRNA MAPKAPK5‐AS1 brings about tumor proliferation by inducing epigenetic silencing of p21.

It enriches the promoter region of p21 with EZH2 and through H3K27me3 modification inhibits its transcription. Ji and colleagues detected lncRNA MAPKAPK5‐AS1 through differential expression analysis and after performing qRT-PCR they reported its high expression in CRC tissues. They further found a direct correlation of elevated expression of MAPKAPK5‐AS1 with larger tumor size and lymph node metastasis [163]. They also demonstrated that in vitro knocking down of MAPKAPK5‐AS1 restored p21 expression which induced growth arrest and raised the apoptosis rate [163].

Similarly, an elevated level of lncRNA HOXA-AS2 in CRC tissues also promotes the growth and proliferation of tumor cells by epigenetically silencing p21. By interacting with EZH2 and LSD1, it recruits these proteins at the promoter region of p21 which brings about H3K27me3 and H3K4me2 modification respectively, hence, silencing p21 transcription [164]. Contrary to MAPKAPK5‐AS1 and HOXA-AS2, the influence of lncRNA BANCR on p21 is not at the mRNA level, instead, it affects its translation. The expression of BANCR is down-regulated in CRC which is responsible for the inhibition of apoptosis tumor cell proliferation both in vivo and in vitro.

Shi et al. detected a direct association between decreased BRAF-activated nonprotein coding RNA (BANCR) expression and reduced p21 protein levels. They treated colon cancer cells with pCDNA-BANCR to induce BANCR expression and found that over-expression of BANCR increased translation of p21 which led to cell cycle arrest and induction of apoptosis [165]. Another proliferation and apoptosis modulating lncRNA FOXP 4‐AS 1 are detected by Li and group which is in vivo responsible for large tumor size and advancement in cancer pathological stage while in vitro it causes rapid cell growth. Mechanistically, its in vitro/in vivo elevated concentrations suppressed apoptosis by down-regulating the expression of p21 and other cell-cycle regulatory proteins. Knock-down of FOXP 4‐AS 1 in DLD‐1, HT‐29 and HCT116 cell lines promoted p21 expression and increased the rate of apoptosis [166]. Similarly, up-regulated lncRNA SNHG6 also promotes tumor progression, invasion and metastasis by bringing about epigenetic silencing of p21 expression. It recruits EZH2 to the promoter region of p21 which then catalyzed methylation of H3 and silence its transcription [167].

LncRNA ATB is another highly expressed lncRNA in CRC tissues that induces cell proliferation by attenuating the expression of p21 and p53. Up-regulation of ATB brings about down-regulation of miR-200c which then inhibits cell apoptosis and causes tumorigenesis [168, 169]. Karimi Mazraehshah and the group reported the association of miR-200c expression with apoptosis in CRC. According to them, miR-200c ectopic expression in SW-48 and HCT-116 facilitates apoptosis by suppressing the expression of the anti-apoptotic protein, BMI1 [170].

Gao et al. demonstrated in lovo and SW-48 cells that the suppression of ATB expression via siRNA boosts miR-200c expression which leads to an elevation in apoptosis rate by inducing p21 and p53 expression [168]. Although these findings suggest the regulatory control of ATB on apoptosis via miR-200c the link between ATB expression and BMI1 expression (which is a direct target of miR-200c) in CRC is currently not backed by any experimental data.

## Overall conclusion

Most studies to date have shown that there is a correlation between lncRNA and CRC. However, it is still difficult to conclude which apoptosis pathway is dominant, and as a result, new mechanistic challenges arise between choosing between targeted or activated blocking. Also, the studies on lncRNA inhibitors are limited. LncRNAs have active participation in promoting tumorigenesis by sabotaging cell growth regulatory processes and molding them in favor of persistent growth. Despite all the advancements, the mortality rate of CRC is consistently increasing with each passing year, mainly because of poor diagnosis and the establishment of chemo-resistance [171]. Evidence has demonstrated the role of lncRNAs in treatment failure which by inhibiting cell apoptotic processes ensures active growth and proliferation of cancer cells. Information that is gathered through years of research has provided somewhat clear reasons for the increased mortality rate from CRC and has also pointed toward a potential solution. But knowledge so far is incomplete. More active investigations in the detection of lncRNAs that could assist in diagnosing cancer at primary stages or could be targeted for suppressing carcinogenesis are needed for an hour. The mechanism of apoptosis is based on sensors that monitor extra- and intracellular signals, effectors such as caspases and regulators, proapoptotic (Bax proteins) or antiapoptotic (Bcl-2). Apoptosis is triggered in two major ways: an extrinsic pathway, mediated by membrane receptors, and an intrinsic or mitochondrial pathway. The extrinsic pathway is initiated by attaching ligands (TNF-α, FAS) to specific membrane receptors, which produce the recruitment and activation of the initiating caspase 8, which in turn will activate the effector caspase 3, responsible for the induction of apoptosis. The intrinsic pathway is triggered in particular by DNA alterations, which can cause the release of proapoptotic factors from the mitochondria, which activate effector caspases 3 and 9.

In cancer, resistance to apoptosis is acquired through various mechanisms that block the extrinsic or intrinsic pathway, but the most important is the loss of proapoptotic regulators through mutations involving suppressor genes and inactivation of Bax or overexpression of the antiapoptotic protein Bcl-2. TRAIL-mediated apoptotic pathway possesses chief significance in shifting weight from normalcy to cancerous. Like in all cancers, TRAIL signaling in CRC is also halted. Although numerous factors have been reported to date that collectively inhibit apoptosis the dysregulation of several endogenous lncRNAs mainly subverts this cellular process. Most of them regulate the expression of miRNAs to exert carcinogenic influences. On the other hand, the role of few lncRNAs (which otherwise could have diagnostic applications) has become controversial due to the existence of mutually exclusive information. For instance, the existence of contradictory data about LINC00152 has created doubts about its authenticity. Similarly, some lncRNAs such as ZFAS1, ATB and FOXP4-AS1 could serve as prognostic markers. Likewise, SNHG6 is another important lncRNA that regulates numerous processes in CRC as evidenced by many studies. Its expression silencing through epigenetic modification or via the action of the drug could be a potential strategy for treating CRC. In conclusion, TRAIL signaling modulating lncRNA is of diagnostic and therapeutic importance. Due to the contribution of some dysregulated-lncRNAs in suppressing apoptosis, their attenuation via synthetic complementary oligomers or using some natural compounds could be a good therapeutic strategy for CRC.

## Data Availability

Yes.
